# Longitudinal evaluation of asymptomatic Leishmania infection in HIV-infected individuals in North-West Ethiopia: A pilot study

**DOI:** 10.1371/journal.pntd.0007765

**Published:** 2019-10-08

**Authors:** Johan van Griensven, Saskia van Henten, Bewketu Mengesha, Mekibib Kassa, Emebet Adem, Mengistu Endris Seid, Saïd Abdellati, Wondimu Asefa, Tesfa Simegn, Degnachew Debasu, Tadfe Bogale, Yonas Gedamu, Dorien Van Den Bossche, Wim Adriaensen, Gert Van der Auwera, Lieselotte Cnops, Florian Vogt, Ermias Diro

**Affiliations:** 1 Department of Clinical Sciences, Institute of Tropical Medicine, Antwerp, Belgium; 2 Medical College, University of Gondar, Gondar, Ethiopia; 3 Medical services, Metema district hospital, Metema, Ethiopia; Academic Medical Centre, NETHERLANDS

## Abstract

**Background:**

In endemic regions, asymptomatic *Leishmania* infection is common. In HIV patients, detection of asymptomatic *Leishmania* infection could potentially identify those at risk of visceral leishmaniasis (VL). However, data on the prevalence, incidence, and determinants of asymptomatic infection, and the risk of VL are lacking.

**Methods:**

We conducted a cross-sectional survey at a single ART centre, followed by a prospective cohort study amongst HIV-infected adults in HIV care in a district hospital in a VL-endemic area in North-West Ethiopia (9/2015-8/2016). Asymptomatic *Leishmania* infection was detected using the direct agglutination test (DAT), rK39-rapid diagnostic test (RDT)), PCR on peripheral blood and the KAtex urine antigen test, and defined as positivity on any *Leishmania* marker. All individuals were followed longitudinally (irrespective of the *Leishmania* test results). Risk factors for asymptomatic *Leishmania* infection were determined using logistic regression.

**Results:**

A total of 534 HIV-infected individuals enrolled in HIV care were included in the study. After excluding 13 patients with a history of VL and an 10 patients with incomplete baseline *Leishmania* tests, 511 were included in analysis. The median age was 38 years (interquartile range (IQR) 30–45), 62.6% were male. The median follow-up time was 12 months (IQR 9–12). No deaths were reported during the study period. Most (95.5%) were on antiretroviral treatment at enrolment, for a median of 52 months (IQR 27–79). The median CD4 count at enrolment was 377 cells/mm3 (IQR 250–518). The baseline prevalence of *Leishmania* infection was 12.8% in males and 4.2% in females. Overall, 7.4% tested positive for rK39, 4.3% for DAT, 0.2% for PCR and 0.2% for KAtex. Independent risk factors for a prevalent infection were male sex (odds ratio (OR) 3.2; 95% confidence intervals (CI) 14–7.0) and concurrent malaria infection (OR 6.1; 95% CI 1.9–18.9). Amongst the 49 prevalent (baseline) infections with further follow-up, the cumulative incidence of losing the *Leishmania* markers by one year was 40.1%. There were 36 incident infections during the course of the study, with a cumulative one-year risk of 9.5%. Only one case of VL was detected during follow-up.

**Conclusions:**

We found a high prevalence of asymptomatic *Leishmania* infection, persisting in most cases. The incidence was more modest and overt VL was rare. Larger and longer studies with more complete follow-up may help to decide whether a test and treat strategy would be justified in this context.

**Trial registration:**

ClinicalTrials.gov NCT02839603

## Author summary

As visceral leishmaniasis (VL) in HIV patients is difficult to treat and associated with high mortality, strategies to detect and treat asymptomatic *Leishmania* infection in HIV patients should be explored. However, data on the prevalence, incidence, determinants of asymptomatic infection and risk of VL are lacking. We conducted a longitudinal study, including HIV-infected adult patients in HIV care in a district hospital in a VL-endemic area in North-West Ethiopia. Asymptomatic *Leishmania* infection was evaluated by *Leishmania* antibody tests (DAT and rK39), urine antigen tests (KAtex) and PCR, and was defined as positivity on any *Leishmania* marker. We also looked for independent risk factors for asymptomatic *Leishmania* infection at study recruitment. A total of 511 patients were included in the analysis. The median age was 38 years, 62.6% were male. The median time of residence in a VL-endemic area was 18 years. Most (95.5%) were on antiretroviral treatment at enrolment, for a median of 52 months. The median CD4 count at enrolment was 377 cells/mm^3^. The baseline prevalence of *Leishmania* infection was 12.8% in males and 4.2% in females. Overall, 7.4% tested positive for rK39, 4.3% for DAT, 0.2% for PCR and 0.2% for KAtex. Independent risk factors for a prevalent infection were male sex and concurrent malaria infection. Amongst the 49 prevalent infections that were present upon enrolment in the study, the probability of losing the *Leishmania* markers by one year was 40.1%. There were 36 new infections during the course of the study, with an overall risk of 9.5% by one year of follow-up. One case of VL was detected during follow-up. In conclusion, we found a high prevalence of asymptomatic *Leishmania* infection, which persisted in most cases. The incidence was more modest. Larger and longer studies would be needed to decide whether a test and treat strategy would be justified in this context.

## Introduction

Visceral leishmaniasis (VL), also called kala-azar, is a vector-born disseminated protozoan infection caused by the *Leishmania donovani* complex, predominantly affecting tissue macrophages. The global annual incidence is estimated at 50,000–90,000 cases. Untreated, overt disease is universally lethal. The anthroponotic form is caused by *L*. *donovani*. This form is prevalent in the Indian subcontinent and East-Africa, mainly in Sudan, South Sudan and Ethiopia [1].

HIV has been identified as one of the emerging challenges for VL control. HIV infection dramatically increases the risk of progression from asymptomatic infection towards disease (VL) and VL accelerates HIV disease progression [2]. Whereas HIV has contributed to the re-emergence of VL in Europe in the 90’s, this problem is now especially severe in North-West Ethiopia, where up to 20–30% of patients with VL are coinfected with HIV [2]. There have also been concerns that some HIV patients infected with *Leishmania* might be more likely to transmit, also at the asymptomatic stage [3–5]. This would hamper VL control efforts.

Management of VL-HIV coinfection remains unsatisfactory, and even more so in East-Africa [6, 7]. Despite increasing availability of antiretroviral treatment (ART) and first-choice VL treatments such as liposomal amphotericin B, mortality remains high. Many patients (up to 50%) fail to clear parasites from infected tissues and/or suffer from recurrent relapses [2, 6, 7]. Given the poor prognosis once *Leishmania* infection has progressed to VL, tackling *L*. *donovani* infection before disease onset looks appealing. There are successful evidence-based examples of World Health Organization (WHO) recommended preventive strategies for other opportunistic infections including a screen and treat strategy recommended for cryptococcal infection, whereby asymptomatic individuals screening positive for early cryptococcal infection receive pre-emptive treatment with fluconazole. Similarly, isoniazid prophylactic therapy is recommended to prevent tuberculosis in individuals with latent infection [8].

Concurrent with the scaling-up of HIV care, there are currently large numbers of HIV-infected individuals living in VL-endemic areas and enrolled in HIV care. In immunocompetent individuals, *Leishmania* infection will usually be controlled by the immune system and treatment of asymptomatic cases is currently not recommended [9]. In contrast, HIV-coinfected, immunosuppressed individuals are likely to have a substantially higher risk of progression to VL and at the same time face a poor prognosis once VL develops [2]. As overt disease is usually preceded by a prolonged period of asymptomatic infection, detectable with different markers of *Leishmania* infection, this period constitutes a window of opportunity for screening strategies integrating markers of *Leishmania* infection to capture those at high risk of VL. Ultimately, this could lead to a screen and treat strategy for VL in HIV-infected individuals living in VL-endemic areas, as is employed for other opportunistic infections [10, 11]. Ideally, this would consist of routine screening with simple *Leishmania* tests, and a cheap and effective treatment for those at high risk of progression, as done for other HIV-associated opportunistic infections.

While we have recently developed the theoretical basis for such a strategy [12], the evidence base for this approach is currently completely lacking. Such a strategy hinges on the hypothesis that asymptomatic *Leishmania* infection is common in HIV-infected individuals living in VL-endemic regions; that markers can be identified predicting the progression to overt disease (VL); and that the asymptomatic period is sufficiently long to intervene before patients progress to VL. While a range of studies on asymptomatic *Leishmania* infection has been conducted in *L donovani*-endemic regions (mainly the Indian subcontinent and East-Africa), these essentially had an epidemiological focus and none focussed on HIV-infected patients. This is of particular importance, as asymptomatic infection is most likely to be of clinical relevance in HIV patients. As a first step to construct the evidence-base, we conducted a longitudinal study in a large HIV patient cohort in a VL-endemic region in North-West Ethiopia to determine the prevalence, incidence and determinants of asymptomatic *Leishmania* infection in HIV-coinfected patients, and the associated risk of VL. The study was set-up as a pilot study to assess the relevance and feasibility of a potential screen and treat strategy in the study area.

## Methods

### Study setting

Ethiopia is an East-African low-income country. It has proportionally the highest VL-HIV burden globally, mostly concentrated in the North-Western part of the country, at the border with Sudan [13]. VL-HIV coinfection is mainly found amongst young seasonal workers, who migrate during the harvest season from VL-nonendemic to VL-endemic regions. During this period, they are likely to be exposed to sandflies carrying *Leishmania*, and are also at risk of contracting HIV. The Metema district hospital, close to the border with Sudan, is located in a VL-endemic region and caters for a population of > 120.000 inhabitants. The hospital HIV program currently has approximately 1500 HIV-infected patients in regular follow-up. HIV guidelines follow national and WHO recommendations [14, 15].

Patients collect ART medication free of charge every two-three months, and CD4 cell count is performed every six months according to national guidelines. During every visit patients are weighed, screened for opportunistic infections and treated for any medical problems if necessary. ART treatment is initiated according to WHO guidelines. During the study period, ART was started when CD4 count reached below 500 cells/μl or when a stage III or IV opportunistic infection had been diagnosed [14]. Self-reported ART adherence was measured at each follow-up visit and defined as good if < 3 pills/month had been missed since the previous visit.

The Metema district hospital is supported by the University of Gondar in terms of VL diagnosis and treatment. The university hosts a Leishmania Research and Treatment Center (LRTC), founded by the Drugs for Neglected Diseases initiative (DND*i*). The center has conducted GCP-compliant VL clinical trials. The laboratory conducts analytical tests and assures quality through accordance with international guidelines.

### Study design, population and recruitment

Between September 2015 and August 2016 we enrolled adult patients under HIV care at the Metema hospital in North-West Ethiopia in a prospective cohort study. HIV patients attending the ART clinic were screened for eligibility consecutively when coming for their regular follow-up visits. To be eligible for inclusion in the study, patients had to be above 18 years of age, with no VL history in the past five years, living in a VL-endemic area for one year or more, and with no intention to move away in the next year. Patients who were diagnosed with VL at recruitment were excluded from the study (see below), as well as those missing a *Leishmania* test at baseline (precluding the determination of status of prevalent infection which was the main outcome). In the analysis for this paper, all individuals with a history of VL were excluded. The target was to purposively recruit 70% males as VL is predominantly seen among males due to their job related exposure [16]. To allow completion of all laboratory procedures the same day, a maximum of ten patients were (consecutively) enrolled daily.

### Sample size calculation

The main outcome measure was prevalence of asymptomatic infection, as a very low prevalence would preclude further studies on a potential screen and treat strategy in the study area. Assuming a prevalence of asymptomatic *Leishmania* infection in HIV patients of 14% [17], a total of 514 patients were required to allow for a required precision of estimates for a prevalence of ± 2.5%. Allowing for a 5% incompleteness of laboratory work-up, a total of 540 patients were to be recruited.

### Study procedures

For all eligible consenting patients, information on the HIV history (HIV diagnosis, WHO staging/opportunistic infections, CD4 counts, initiation of ART), degree of VL exposure (sleeping outside, VL cases in the household or neighborhood, years living in VL endemic area), and current medical condition was collected, combined with a systematic clinical evaluation. As the study focused on asymptomatic *Leishmania* infection, all individuals meeting the national clinical case definition (fever >2 weeks with weight loss and/or splenomegaly in patients from a VL-endemic area) for VL at baseline were immediately screened using the rK39 rapid diagnostic test (RDT) as per national guideline. rK39 positive patients meeting the clinical case definition at the time of recruitment were excluded from the study. For the rest of the participants blood, urine and stool samples were collected at baseline (M0) and thereafter every three months up until one year (M3, M6, M9, M12) or until closure of the study. A maximum follow-up period of one year was foreseen for each patient (irrespective of the results of the *Leishmania* markers). In January 2017, the accumulating evidence of the pilot study suggested the incidence of VL to be very low in the study population. Consequently, the study site was closed. With the study closing six months after the last patient was recruited, the individual follow-up time ranged from six to 12 months. There were 380 (74.4%) patients that reached twelve months of follow-up, 105 (20.5%) reaching nine months of follow-up and 26 (5.1%) six months of follow-up. The study is registered at ClinicalTrials.gov with NCT02839603 as identifier.

### Laboratory assays and quality control

The following tests were done systematically: complete blood count (CBC) (Mindray BC-3000 PlusMindray, China), CD4 count (BD FACSCount, Beckton Dickinson, USA), stool microscopy on fresh samples for intestinal parasites, microscopy for malaria detection in blood, *Leishmania* serological tests (the direct agglutination test (DAT, Institute of Tropical Medicine—Antwerp (ITM-A), Belgium) and a rK39 RDT (IT-LEISH, BioRad, USA), a *Leishmania* antigen test on urine (KAtex, Kalon Biological Ltd, UK)[18] and real-time PCR on whole blood. The first four tests and the rK39 RDT were performed locally at the Metema hospital. The sensitivity of serological tests to detect VL is reportedly slightly lower in HIV patients in East-Africa [19].

CBC and CD4 count were done in accordance with manufacturer instructions. Stool examination by direct microscopy was performed on a wet mount from freshly collected samples at magnifications of 100x and 400x. Thick and thin smears for malaria microscopy were prepared from venous EDTA blood. Thin films were fixated with methanol, both were stained with Giemsa pH 7.2 and examined by light microscopy at a magnification of 100x with oil immersion. The rK39 RDT was done using serum, following the instructions of the manufacturer.

Samples for DAT, KAtex and PCR were transported in cold chain boxes to LRTC in Gondar for storage and further analysis. For DAT, a freeze-dried version of the DAT antigen composed of fixed, trypsin-treated and stained promastigotes of *L*. *donovani* prepared in the ITM-A was used [20]. DAT testing was done on serum as previously described [20]. The presence of leishmanial antigens in the urine was assessed using the KAtex urine antigen test according to manufacturer’s instructions (Kalon Biological Ltd).

DNA was extracted from 200 or 300 μl whole blood, with the QIAamp DNA mini kit (Qiagen Benelux, Venlo, The Netherlands) or the Maxwell LEV DNA kit on the automated Maxwell 16 platform (Promega, The Netherlands) respectively. Elution was done in 100 (QIAamp) or 65 (Maxwell) μl, following validation [13]. *Leishmania* was detected with a PCR targeting either the small subunit (18S) rDNA [14, Deborggraeve et al], or kDNA minicircles [15, Mary et al.], or both. A real-time hydroylysis probe-based PCR was used, with the laboratory-developed probe FAM-CTGGTCGTCCCGTCCATGTCGGATT-BHQ1-ZEN for the 18S rDNA PCR. The primers and kDNA probe were as in aforementioned papers. PCRs were done with the HotStarTaq Master mix kit (Qiagen) in a total volume of 25 μl containing 1x mastermix, 0.4 (18S rDNA) or 0.6 μM (kDNA) of each primer, 0.1 (18S rDNA) or 0.4 μM (kDNA) of the hydrolysis probe (IDT), a total of 4.5 (18 rDNA) or 1.5 mM (kDNA) MgCl_2_, 0.1% BSA, and 5 μl DNA. 40 (18S rDNA) or 50 (kDNA) cycles were ran in the RotorGeneQ (Qiagen), after an initial denaturation and enzyme activation step of 15 min at 95°C. Cycles consisted of 5 sec at 95°C denaturation, 20 sec at 58°C annealing, and 30 sec at 72°C polymerization. Negative extraction (PBS) and no-template PCR controls were used to monitor contamination, and positive PCR controls were included to check for PCR efficiency.

Quality of results was assured through internal and/or external quality controls. Manufacturer’s internal quality controls were used for CBC and CD4 count. As these controls were not always readily available in the region, a comparison of CBC and CD4 count with LRTC was performed regularly. For KAtex and DAT, internal controls delivered with the kit were included per batch of tests. One known rK39 RDT positive and one negative sample were tested weekly. External quality control panels were prepared by the ITM and analyzed frequently for rK39RDT, DAT, KAtex, stool and malaria microscopy. For PCR, DNA extracted at LRTC, were re-tested at ITM as external quality control.

### Data collection

Study-related information was collected using a clinical and a laboratory case report form. There was ongoing monitoring of the quality of the data collected; all staff were trained on the protocol, GCP, laboratory quality management system, and data collection procedures. Data entry was done in Epi Info 3.5.4 (CDC, USA). Data quality checks were done before locking the final database.

### Data analysis

The main outcome measure was asymptomatic *Leishmania* infection. In line with the vast majority of previous studies, this was defined as positivity on at least one of the *Leishmania* markers (detection of anti-leishmanial antibodies by rK39 RDT and DAT, of *Leishmania* DNA by PCR, and of *Leishmania* antigen in urine by the KAtex test) [20–24].

Age was grouped per decade as commonly done in similar studies, as was time lived in a VL-endemic area. Time since ART initiation was grouped with a focus on the first year of ART, since the risk for HIV-associated opportunistic infections generally starts declining after the first year of ART [25]. CD4 was grouped according to thresholds used to start ART and cotrimoxazole (>500, 350–500, <350 cells/μL). Body mass index (BMI) was grouped according to internationally used thresholds in the categories severely to moderately malnourished (BMI<17 kg/m^2^), mildly malnourished (BMI ≥17 and <18.5 kg/m^2^), no malnutrion (BMI ≥ 18.5 kg/m^2^).

A DAT titer ≥ 1/1600 was used to define asymptomatic infection, as used before [20]. DAT-positive cases were further subdivided as moderately (titer ≥ 1/1600 & < 1.25600) and strongly seropositive (titer ≥ 1/25600). KAtex results were scored as 0, 1+, 2+ or 3+ according to the degree of agglutination as per manufacturer’s instructions. Any degree of positive grading was considered as a positive test. The rK39 RDT was interpreted as positive or negative according to the manufacturer’s instructions. PCR results were scored as positive, negative, or indeterminate if a high cycle threshold (Ct) value was observed that could not be reproduced in repeated PCRs. Hence the signal seen in such indeterminate reaction could have been caused by a contamination.

The study population was described using median and interquartile ranges (IQR), and counts and proportions. The prevalence of asymptomatic *Leishmania* infection in HIV patients was determined as a proportion with 95% Wilson confidence intervals (CI). In addition, prevalence of asymptomatic infection was calculated for every marker separately. For the univariate analysis, odds ratios (ORs) with 95% CIs were calculated for selected potential risk factors. *P*-values below 0.05 from Likelihood Ratio Tests (LRT) were considered statistically significant. To identify risk factors associated with asymptomatic *Leishmania* infection, a multivariate model was built based on stepwise backwards deletion starting with the weakest univariate exposure-outcome association at each variable group separately. Variables were grouped by socio-demographic characteristics, factors related to reservoir and vector exposure, and clinical factors. For each group, exposure variables with a *p*-value below 0.1 were kept in the model. In the final model, the independent associations of retained exposure variables were tested using LRTs.

The incidence rate of asymptomatic infection was calculated by dividing the number of incident infections over the total person follow-up time among patients without infection at baseline. Follow-up time started on the date of enrolment until the date of detection of an asymptomatic infection, or the latest study visit for the remainder. Additionally, we calculated the cumulative incidence of these outcomes at various time points using Kaplan-Meier methods. To allow comparison with other studies, analysis was repeated using only the serological markers to define asymptomatic infection. We also calculated the rate of reversion to negative *Leishmania* markers in patients with a prevalent infection, with follow-up time censored at the date of the first negative results (reversion) of all *Leishmania* markers amongst those with a prevalent infection at enrolment, or the latest study visit for those remaining with positive markers. Data analysis was done using STATA 14.0 (STATACorp LP, USA).

### Ethics

The protocol was approved by the institutional review board of the University of Gondar, the ITM institutional review board of the Institute of Tropical Medicine, Antwerp and the Ethics committee of the University of Antwerp, Belgium. All patients gave written informed consent.

## Results

### Patient characteristics

Between September 2015 and August 2016, a total of 534 HIV-infected individuals enrolled in HIV care were included in the study. After excluding 13 patients with a history of VL and 10 patients with incomplete baseline *Leishmania* tests, 511 were included in analysis. The median follow-up time was 12 months (IQR 9–12). No deaths were reported during the study period. The majority of patients was male (62.6%—study target was 70%); with a median age of 38 years (IQR 30–45). Most were farmers or daily laborers (37.1% and 22.4%, respectively), without formal education (57.4%) but literate (75.5%), and married (51.7%). More than half (55.0%) of patients slept outside regularly, 3.0% had a VL case in the household ever, while 5.5% ever had a VL case in the neighbourhood. The median time of residence in a VL-endemic area was 18 years (IQR 10–25).

The majority of patients (95.5%) were on ART, with a median of 52.2 months on treatment (IQR 27.3–79.5). At study enrolment, the median CD4 count was 377 cells/mm3 (IQR 250–518) and the median BMI was 19.7 kg/m^2^ (IQR 18.2–21.5). Self-reported ART adherence during follow-up was generally good with > 90% of individuals reporting good ART adherence (missing <3 tablets/month since the previous visit). Intestinal parasitosis was present in 20.5%, and malaria infection was detected in blood smears in 2.9% (Table 1).

**Table 1:** Patient baseline characteristics (Ethiopia, 2015–2016).

	Total (N = 511)	
	N	%
**Socio-demographic**		
Men	320	62.6
Age in years, median (IQR)	38	30–45
Literate patients	382	75.5
**Occupation of patient**		
Farmer	189	37.1
Daily labourer	114	22.4
Merchant	77	15.1
Housewife	77	15.1
Other	52	10.2
**Educational level of patient**		
None	292	57.4
Primary	163	32.0
Secondary/tertiary	54	10.6
**Marital status of patient**		
Married	263	51.5
Divorced	152	29.8
Widowed	53	10.4
Single	41	8.0
**Exposure**		
Patients sleeps outside	277	55.0
VL in patients’ household	15	3.0
VL in patients’ neighborhood	28	5.5
Years lived in endemic area, median (IQR)	18	10–25
**Clinical**		
On ART	484	95.5
Months since ART initiation, median (IQR)	52.2	27.3–79.5
Any opportunistic infection(s) before	358	70.8
WHO clinical stage		
I/II	211	41.5
III	259	51.0
IV	38	7.5
CD4 count, median (IQR); (cells/μl)	377	250–518
Hemoglobin, median (IQR); (g/dL)	14.4	13.0–15.6
White blood cells, median (IQR); (cells/mm^3)^	5.1	3.9–6.6
Platelets, median (IQR); (cells/mm^3)^	213	167–269
Patients with chronic comorbidities	7	1.4
Patients with intestinal parasitosis^a^	103	20.5
Patients with malaria infection	15	2.9
BMI of patients, median (IQR); kg/m^2^	19.7	18.2–21.5

ART: antiretroviral treatment; BMI: body mass index; IQR: interquartile range; VL: visceral leishmaniasis; WHO: World Health Organization

^a^ G. lamblia (n = 49), E. histolytica (n = 48), hookworm (n = 8), S. stercoralis (n = 5), S. mansoni (n = 5), H. nana (n = 2), A. lumbricoides (n = 2)

### Prevalence of and factors associated with asymptomatic *Leishmania* infection and reversion of baseline infection markers

The availability and results of tests for asymptomatic infection at the different time points is given in S1 Table. The prevalence of asymptomatic *Leishmania* infection at baseline was 12.8% in males and 4.2% in females, with the highest prevalence (17.9%) found in males aged between 28 and 38 years old (Table 2).

**Table 2:** Prevalence of asymptomatic *Leishmania* infections amongst HIV infected individuals living in a VL endemic area, North-West Ethiopia (n = 511).

	Males; n/N (row %)	Females; n/N (row %)
Age (years)		
18–28	4/34 (11.8)	0/63 (0)
28–38	19/106 (17.9)	3/71 (4.2)
38–48	12/118 (10.2)	2/34 (5.9)
≥ 48	6/62 (9.7)	3/23 (13.0)
Total	41/320 (12.8)	8/191 (4.2)

**Fig 1:**
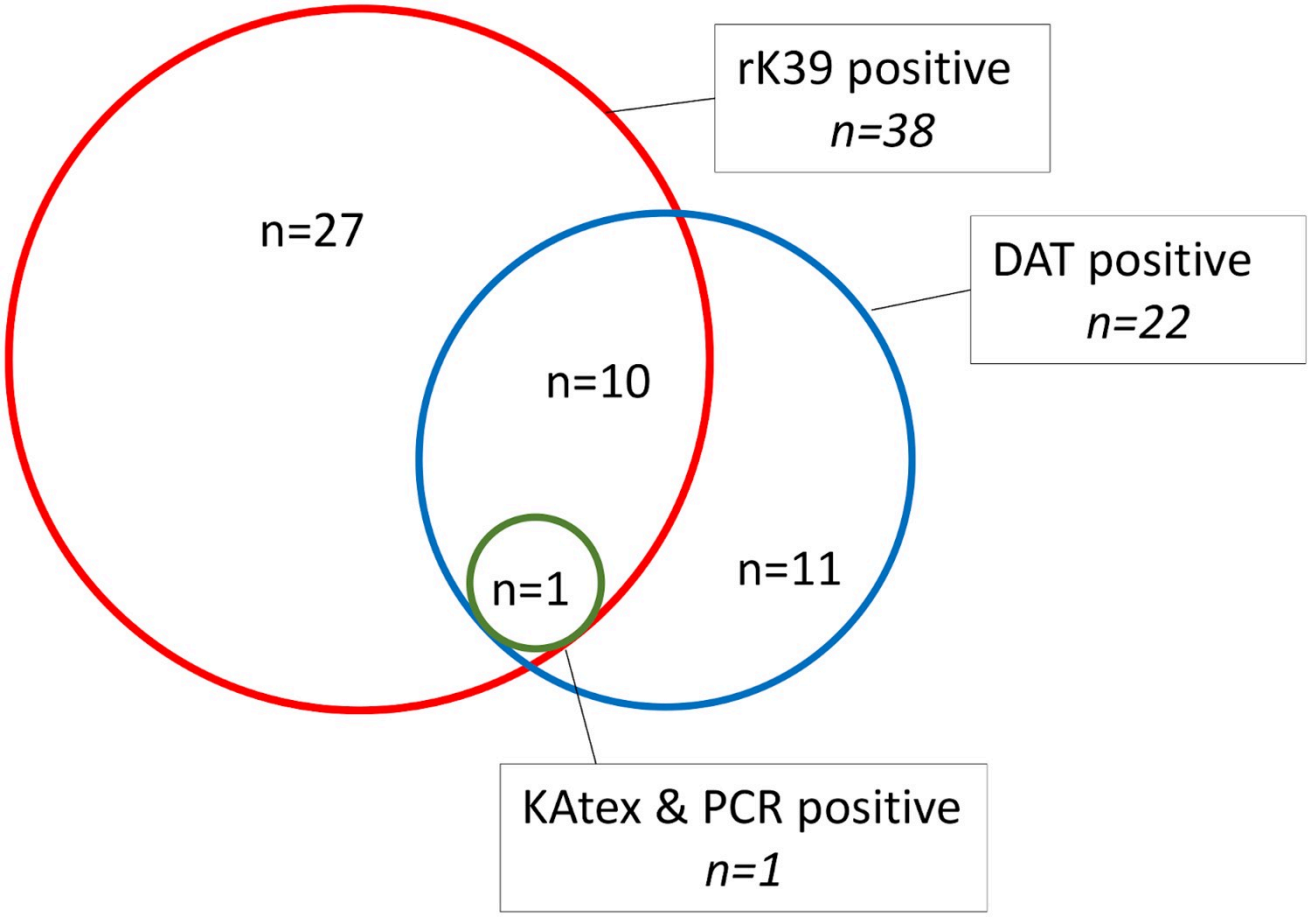
Type of positive *Leishmania* markers at the time of recruitment in the study (prevalence). DAT: direct agglutination test; PCR: polymerase chain reaction.

At the time of recruitment, 7.4% of patients tested positive on the rK39 RDT, 4.3% on DAT, 0.2% on PCR and 0.2% on KAtex. A total of 27 (55.1%) asymptomatically infected patients were positive only on the rK39 RDT, while 10 (20.4%) were positive on both rK39 and DAT and 11 (22.4%) tested positive only on DAT. One patient tested positive at baseline for all four *Leishmania* markers (Fig 1).

Risk factors identified in univariate analysis were being male, sleeping outside and having malaria (Table 3).

**Table 3:** Factors associated with prevalent (baseline) asymptomatic *Leishmania* infection in HIV-infected individuals (Ethiopia 2015–2016).

Patient baseline characteristics	Asymptomatic infection^a^ (N = 49)		No infection (N = 462)		Univariate analysis		
	n	%	N	%	OR	95% CI	p-value
**Socio-demographic**							
**Male sex**	41	83.7	279	60.4	3.4	1.5–7.3	<0.001
**Age of patient (years)**							
[18–28)	4	8.2	93	20.1	1		0.121
[28–38)	22	44.9	155	33.6	3.3	1.1–9.9	
[38–48)	14	28.6	138	29.9	2.4	0.8–7.4	
≥48	9	18.4	76	16.5	2.8	0.8–9.3	
**Occupation of patient**							
Farmer	25	51.0	164	35.5	2.2	0.8–6.0	
Daily labourer	16	32.7	98	21.2	2.4	0.8–6.7	
Merchant	0	0.0	77	16.7	-		
Housewife	5	10.2	72	15.6	1		0.1436
Other	3	6.1	49	10.6	0.9	0.2–3.9	
**Marital status**							
Married	25	51.0	238	51.5	1		0.092
Divorced	16	32.7	136	29.4	1.1	0.6–2.2	
Widowed	1	2.0	52	11.3	0.2	0.0–1.4	
Single	6	12.2	35	7.6	1.6	0.6–4.3	
**Exposure**							
**Patient sleeps outside**	34	69.4	243	52.6	2.1	1.1–4.1	0.018
**VL in patients’ household**	1	2.0	14	3.0	0.7	0.1–5.2	0.678
**VL in patients’ neighbourhood**	2	4.1	26	5.6	0.7	0.2–3.1	0.626
**Time lived in endemic area (years)**							
<10	12	24.5	97	21.0	1		0.688
10 to <20	15	30.6	157	34.0	0.8	0.3–1.7	
20 to <30	13	26.5	143	31.0	0.7	0.3–1.7	
≥30	9	18.4	61	13.2	1.2	0.5–3.0	
**Clinical**							
**Patient is not on ART**	4	8.2	19	4.1	2.2	0.7–6.6	0.212
**Patient has intestinal parasitosis**	15	30.6	88	19.1	1.8	1.0–3.5	0.077
**Patient has malaria infection**	6	12.2	9	2.0	7.0	2.4–20.6	0.001
WHO stage							
I	12	24.5	116	25.1	1		0.436
II	12	24.5	71	15.4	1.6	0.7–3.8	
III	21	42.9	238	51.5	0.9	0.4–1.8	
IV	4	8.2	34	7.4	1.1	0.3–3.8	
**Time since ART initiation (months)**							
<3 or not on ART	6	12.2	34	7.4	2.0	0.8–5.1	
3 to <12	1	2.0	30	6.5	0.3	0.0–2.8	
12 to <24	9	18.4	48	10.4	1.6	0.9–4.7	
≥24	31	63.3	347	75.1	1		0.109
**CD4 cell count of patient (cells/μL)**							
<100	2	4.1	32	6.9	0.9	0.2–4.5	
100 to <350	21	42.9	173	37.5	1.8	0.8–4.0	
350 to <500	15	32.7	121	26.2	2.0	0.83–4.59	
≥500	9	18.4	133	28.8	1		0.317
**BMI of patient (kg/m^2^)**							
<17.0	5	10.2	52	11.3	0.8	0.3–2.2	
17.0-<18.5	10	20.4	85	18.4	1.0	0.5–2.2	
18.5-<25	33	67.4	287	62.1	1		0.380
> = 25.0	1	2.0	36	7.8	0.2	0.0–1.8	

ART: antiretroviral treatment; BMI: body mass index; VL: visceral leishmaniasis; WHO: World Health Organization

Multivariate analysis showed the risk of asymptomatic *Leishmania* infection to be increased for male patients (OR 3.2, 95% CI 1.4–7.0, p = 0.002), and for patients with malaria infection (OR 6.1, 95% CI 1.9–18.9, p = 0.004) (Table 4).

**Table 4:** Multivariable risk factor analysis for asymptomatic Leishmania infection in HIV-infected individuals (Ethiopia 2015–2016).

Variable	OR	95% CI	p-value
Male sex	3.2	1.4–7.0	**0.002**
Time on ART (months)			
<3	2.4	0.9–6.3	
3–12	0.3	0.0–2.5	
12–24	2.0	0.8–4.6	
>24	1		0.081
Malaria infection	6.1	1.9–18.9	**0.004**

ART: antiretroviral treatment; CI: confidence interval; OR: odds ratio.

Of the 49 cases with prevalent infection at baseline, 46 had follow-up samples available, with a total patient follow-up time of 33 person-years. Of these, 16 reverted to negative *Leishmania* markers during follow-up, with a reversion rate of 48.5/100 person years. The estimated probability of reversion was 26.3% by six months, and 40.1% by 12 months (Table 5).

**Table 5:** Incidence of reversion to negative *Leishmania* markers amongst HIV (+) individuals with a prevalent asymptomatic Leishmania infection and incidence of new *Leishmania* infections in the remaining individuals.

Type of event	Pt no	No of events	Total FU time in person years	Rate/100 pt years	Cumulative incidence of event at different months; % (95% CI)^a^			
					M3	M6	M9	M12
**Reversion of prevalent infection**								
Asymptomatic infection (any marker)	46	16	33	48.5	17.4	26.3	28.8	40.1
rK39/DAT	46	16	33	48.5	17.4	26.3	28.8	40.1
**Incidence of (new) infection**								
Asymptomatic infection (any marker)	426	36	353	10.2	4.2	5.0	7.8	9.5
rK39 RDT/DAT	426	16	358	4.5	1.6	2.1	3.2	4.4

^a^ Kaplan-Meier estimates

CI: confidence interval; DAT: direct agglutination test; FU: follow up; pt: patient, RDT: rapid diagnostic test

Restricting the analysis to serological tests only (rK39 or DAT reverting to negative amongst those with positive serological tests at baseline—seroreversion) did not alter the results.

### Incidence of asymptomatic *Leishmania* infection

Of the 462 individuals testing negative on all *Leishmania* markers at enrolment (*Leishmania*-negative individuals), 426 had follow-up samples available, with a total follow-up time of 353 person-years. Out of these, 36 developed asymptomatic infection, yielding an incidence rate of 10.2/100 person-years. The estimated cumulative incidence was 5.0% by six months, and 9.5% by 12 months (Table 5). When analysis was restricted to serological tests (rK39 RDT and DAT) (rK39/DAT seroconversion), the incidence rate was 4.5/100 person-years. Of the 36 incident cases, five tested positive on rK39 only (rK39 seroconversion), 10 on DAT only, one on both DAT and rK39 and 20 on PCR only (Table 6).

**Table 6:** Incidence and pattern of asymptomatic *Leishmania* infections amongst HIV infected individuals living in a VL endemic area, North-West Ethiopia (n = 426).

	M3	M6	M9	M12	Total
Number tested	384	369	351	226	-
Asymptomatic infection					
Incident (at time-point)	18	3	10	5	36
Total (cumulative)	18	21	31	36	36
Pattern of incident infections					
DAT (+)	5	1	2	2	10
rK39 (+)	1	1	2	1	5
rK39 & DAT (+)	1	0	0	0	1
PCR (+)	11	1	6	2	20

DAT: direct agglutination test; PCR: polymerase chain reaction

Amongst all individuals enrolled in the study, one was clinically diagnosed with VL at month nine of follow-up. The patient was on ART at enrolment but with low CD4 cell counts (41 cells/mm3). At enrolment, at three months and at six months, there were no symptoms or signs suggestive of VL (no fever, no hepatosplenomegaly). Self-reported ART adherence was good at each follow-up visit. At month nine, the patient developed fever and hepatosplenomegaly. While it was the intention to refer the patient to the LRTC for tissue aspiration, transport was not possible due to a violent conflict around the study site at that point in time. With the rK39 test additionally being positive, a clinical diagnosis of VL was made. Treatment with liposomal amphotericin B was started and the patient was discharged clinically cured. In retrospect, the markers during follow-up suggested a prolonged phase of asymptomatic infection during the nine months before the patient became symptomatic (see Table 7), with positive serological tests, parasite antigen detected in the urine and relatively low Ct-values on PCR corresponding to relative high levels of parasite DNA (Table 7).

**Table 7:** Evolution of *Leishmania* markers in an HIV-infected individual developing VL after nine months of follow-up.

Time point	rK39 RDT	DAT titer	PCR Ct value	Urine antigen	CD4 count	Fever	HSM
M0	Pos	>1/204800	28.2	2+	26	0	0
M3	Pos	>1/204800	20.3	2+	25	0	0
M6	Pos	>1/204800	21.4	2+	35	0	0
M9	Pos	>1/204800	17.2	2+	35	+	+

Ct: cycle threshold; DAT: direct agglutination test; HSM: hepatosplenomegaly; PCR: polymerase chain reaction, RDT: rapid diagnostic test

## Discussion

To the best of our knowledge, this is the first longitudinal study on asymptomatic *Leishmania* infection in individuals enrolled in HIV care in a *L donovani*-endemic area. Asymptomatic *Leishmania* infection was documented in 12.8% in males and in 4.2% in females, using DAT, rK39 RDT, KAtex urine antigen and PCR on peripheral blood as markers of infection. Male sex and presence of concurrent malaria infection were identified as risk factors. A positive rK39 test was the most common marker of asymptomatic infection. The probability of reversion to negative markers was estimated at 40.1% by one year. There were 36 incident infections, with a one year cumulative incidence of 9.5%.

Only a few studies on asymptomatic *Leishmania* infection have been conducted in Ethiopia, and none in HIV-infected individuals. A recent study using quantitative real-time kDNA PCR found a positive result in 14% [17]. Another study in children in the Amhara region found a prevalence of 10% [23]. Of interest was that in the latter study a relatively large proportion was only detected by *Leishmania* skin testing (LST). None of these studies assessed the association with malaria. The exact mechanism behind the observed association in our study remains to be defined. Possible reasons include the fact that both are transmitted by vectors biting at night or that concurrent malaria infection can lead to false-positive serological tests [26]. Whether malaria infection could increase susceptibility for *Leishmania* infection (or vice-versa) remains to be assessed.

The estimated reversion rate was 40.1% by 12 months. In India, the probability or seroreversion has varied between 0 and 87% by 12 months [20, 27, 28]. In Ethiopia, a recent study amongst the general population in northern Ethiopia reported that 71% of DAT-positive individuals seroreverted by 12 months [29]. High rates of LST reversion have also been observed in the South of Ethiopia [11].

The cumulative incidence of asymptomatic infection was estimated at 9.5% by 12 months in our study, with a rate of 10.2/100 patient years. This is generally comparable with findings in HIV-negative individuals in India, Sudan and older studies from Ethiopia [11, 30, 31], although differences exist in the types of tests used to detect asymptomatic infections. Moreover, when comparing our findings with other studies, the specific selection criteria in our study need to be kept in mind. We purposively over-sampled male patients, as they are at higher risk of VL. As this was a pilot study aiming to longitudinally monitor *Leishmania* markers, we selectively targeted patients most likely to remain in follow-up (“stable residents”). For this reason, recent and/or temporary residents were excluded. As this group is likely to contain migrant workers—whom often come from non-endemic regions and have relatively high levels of exposure while working on the fields—our data might underestimate the prevalence and incidence for the entire group of HIV-infected individuals who visited the ART clinic during the study period.

This probably also contributed to the fact that our study population was generally on ART for prolonged periods, with fairly high CD4 counts. While this stable population allowed for longitudinal monitoring of the different *Leishmania* markers, it could also explain why only one VL diagnosis was made. The fact that Metema is hypo-endemic for VL could also contribute to that finding. We admit however that, as VL can present atypically in HIV patients [32], we can not exclude that some cases might have been missed. Irrespective, our larger cohort (the PreLeisH study, https://clinicaltrials.gov/ct2/show/NCT03013673), with adapted inclusion criteria and *Leishmania* markers (including also rK39 ELISA), recruiting more mobile patients in a nearby VL-hyperendemic area (Abderafi, North West Ethiopia) will hopefully give more insights in the next years. Pending these results, the findings from the study in Metema suggest that the risk of VL in HIV patients stable on ART with good CD4 counts is low. Public health priority in such a setting would rather be to target interventions to those patients that have HIV diagnosed concurrent with a VL diagnosis.

A positive serology was the most common marker of asymptomatic infection. In line with other studies [22, 23], there was only a moderate degree of overlap between the two serological methods used, and combining both substantially increased the yield of seropositivity. With only five positive tests for KAtex, it would be interesting to evaluate recently developed novel urine antigen tests with higher sensitivity [33]. Only one PCR result was positive at baseline, in contrast with a positivity rate of 14% in a study conducted in a VL-hyperendemic area in Tigray [17]. However, that study mostly reported low levels of parasite DNA in the peripheral blood (high Ct-values) and 40% of these could not be confirmed on repeat testing. On the other hand, PCR positive results were not uncommon during follow-up, at least at month three and nine of follow-up. Whether this relates to seasonality requires further study. Future studies should assess whether HIV patients with asymptomatic *Leishmania* infection are more likely to transmit the infection, as has been observed in Europe [3].

There are a number of important limitations to the study. First, as adding a test for *Leishmania*-specific cellular immunity (e.g. LST) would likely have allowed detection of additional infections, our estimates are probably underestimated. We did, however, not use the LST, as no good manufacturing practices (GMP)-compliant products were available during the study period. Missing samples further contribute to the underestimation of incident infections. Similarly, the performance of serological tests for VL diagnosis is sub-optimal in East-Africa and potentially further impaired by HIV infection, impacting the sensitivity of serological tests to detect asymptomatic infections. Irrespective, a key issue for all studies on asymptomatic infection is the lack of a gold standard, hence it is impossible to determine which tests have the highest sensitivity or specificity, or to know the true prevalence and incidence of asymptomatic infection. As some of the Leishmania tests used likely have a low sensitivity and suboptimal specificity to detect asymptomatic infection, false positive results can occur, particularly for the serological tests. Future studies could explore alternative (more specific) definitions of asymptomatic infection such as requiring at least two positive tests to define asymptomatic infection. Particularly for serological tests, it remains to be defined whether these really can be used to define asymptomatic infections or rather indicate past or present exposure. Our study might also be subject to selection bias as our study was not representative of the HIV-infected population of the region. However, the ultimate aim of the larger (PreLeish) cohort study in which this analysis was embedded, is to design a screen and treat strategy for HIV patients enrolled in HIV care. Additionally, our data only apply to HIV-positive residents of the Metema district in Northwestern Ethiopia, and not to migrant workers traveling to this region during the harvest season. The study was underpowered as we included slightly less patients than foreseen. Finally, no reliable data on co-morbidities such as tuberculosis could be collected.

In conclusion, this is the first study on asymptomatic *Leishmania* infection in individuals enrolled in HIV care in a *L donovani*-endemic area. The prevalence of infection was 12.8% in males and 4.2% in females, and highest in young male farmers and daily laborers. The incidence was comparable to other studies. Most patients were on ART for many years, with high CD4 counts. Only one patient developed VL during follow-up. Larger and longer studies with more complete follow-up may help to decide whether a test and treat strategy would be justified in this context.

## Supporting information

S1 Checklist STROBE checklist. (DOC)

S1 Table Markers of asymptomatic Leishmania infection at different time points during follow-up (Ethiopia 2015–2016). DAT: direct agglutination test; PCR: polymerase chain reaction; RDT: rapid diagnostic test.
(DOCX)
