# Supplementary material for: Longitudinal evaluation of asymptomatic Leishmania infection in HIV-infected individuals in North-West Ethiopia: A pilot study
Source: PLoS Negl Trop Dis. 2019 Oct 8;13(10):e0007765. doi: 10.1371/journal.pntd.0007765 (PMC6799935; doi:10.1371/journal.pntd.0007765)
Supplement: S1 Table — DAT: direct agglutination test; PCR: polymerase chain reaction; RDT: rapid diagnostic test. (DOCX) [file pntd.0007765.s002.docx]

| ***Leishmania* infection markers** | **at M0** | | | **at M3** | | **at M6** | | **at M9** | | **at M12** | | |
| --- | --- | --- | --- | --- | --- | --- | --- | --- | --- | --- | --- | --- |
|  | **N** | | **%** | **n** | **%** | **n** | **%** | **n** | **%** | **n** | **%** | |
| **Visits that took place** | 511 (100) | | | 428 (83.8) | | 433 (84.7) | | 405 (79.3)^a^ | | 303 (59.3)^b^ | | |
| **Asymptomatic infection^a^** | N=511 | | | N=405 | | N=396 | | N=353 | | N=275 | | |
| Yes | 49 | | 9.6 | 47 | 11.6 | 37 | 9.3 | 39 | 11.1 | 28 | | 10.2 |
| No | 462 | | 90.4 | 358 | 88.4 | 359 | 90.7 | 314 | 89.0 | 247 | | 89.8 |
| *Missing* | *(0)* | | *(0)* | *(23)* | *(5.4)* | *(37)* | *(8.6)* | *(52)* | *(12.8)* | *(28)* | | *(9.2)* |
| **rK39-RDT** | N=511 | | | N=419 | | N=410 | | N=372 | | N=284 | | |
| Positive | 38 | | 7.4 | 27 | 6.4 | 26 | 6.3 | 27 | 7.3 | 18 | | 6.3 |
| Negative | 473 | | 92.6 | 392 | 93.6 | 384 | 93.7 | 345 | 92.7 | 266 | | 93.7 |
| *Missing* | *(0)* | | *(0)* | *(9)* | *(2.1)* | *(23)* | *(5.3)* | *(33)* | *(8.2)* | *(19)* | | *(6.3)* |
| **KAtex** | N=511 | | | N=410 | | N=414 | | N=359 | | N=282 | | |
| Positive | 1 | | 0.2 | 1 | 0.2 | 2 | 0.5 | 1 | 0.3 | 0 | | 0.0 |
| 1 | 0 | | 0.0^c^ | 0 | 0.0^c^ | 0 | 0.0^c^ | 0 | 0.0^c^ | 0 | | 0.0^c^ |
| 2 | 1 | | 100.0 ^c^ | 1 | 100.0^c^ | 2 | 100.0^c^ | 1 | 100.0^c^ | 0 | | 0.0^c^ |
| 3 | 0 | | 0.0^c^ | 0 | 0.0^c^ | 0 | 0.0^c^ | 0 | 0.0^c^ | 0 | | 0.0^c^ |
| Negative | 510 | | 99.8 | 409 | 99.8 | 412 | 99.5 | 358 | 99.7 | 282 | | 100.0 |
| *Missing* | *(0)* | | *(0)* | *(18)* | *(4.2)* | *(19)* | *(4.4)* | *(46)* | *(11.4)* | *(21)* | | *(6.9)* |
| **DAT** | N=511 | | | N=415 | | N=413 | | N=360 | | N=282 | | |
| Reactive | 32 | | 6.3 | 21 | 5.1 | 25 | 6.1 | 20 | 5.6 | 16 | | 5.7 |
| Reactive, titre missing | 0 | | 0.0^c^ | 0 | 0.0^c^ | 1 | 4.0^c^ | 1 | 5.0^c^ | 0 | | 0.0^c^ |
| 1:200 | 1 | | 3.1^c^ | 0 | 0.0^c^ | 0 | 0.0^c^ | 0 | 0.0^c^ | 0 | | 0.0^c^ |
| 1:400 | 1 | | 3.1^c^ | 0 | 0.0^c^ | 3 | 12.0^c^ | 2 | 10.0^c^ | 0 | | 0.0^c^ |
| 1:800 | 8 | | 25.0^c^ | 1 | 4.8^c^ | 5 | 20.0 ^c^ | 5 | 25.0^c^ | 5 | | 31.3^c^ |
| 1:1600 | 7 | | 21.9^c^ | 4 | 19.0^c^ | 10 | 40.0^c^ | 5 | 25.0^c^ | 6 | | 37.5^c^ |
| 1:3200 | 6 | | 18.8^c^ | 8 | 38.1^c^ | 3 | 12.0^c^ | 3 | 15.0^c^ | 2 | | 12.5^c^ |
| 1:6400 | 0 | | 0.0^c^ | 1 | 4.8^c^ | 0 | 0.0^c^ | 1 | 5.0^c^ | 1 | | 6.3^c^ |
| 1:12800 | 3 | | 9.4^c^ | 2 | 9.5^c^ | 2 | 8.0^c^ | 0 | 0.0^c^ | 1 | | 6.3^c^ |
| ≥1:25600 | 6 | | 18.8^c^ | 5 | 23.8^c^ | 6 | 24.^c^ | 3 | 15.0^c^ | 1 | | 6.3^c^ |
| Negative | 479 | | 93.7 | 394 | 94.9 | 403 | 93.1 | 340 | 94.4 | 266 | | 94.3 |
| *Missing* | *(0)* | | *(0)* | (13) | *(3.0)* | *(20)* | *(4.6)* | *(45)* | *(11.1)* | *(21)* | | *(6.9)* |
| **PCR** | N=511 | | | N=416 | | N=410 | | N=365 | | N=283 | | |
| Positive | 1 | | 0.2 | 12 | 2.9 | 2 | 0.5 | 7 | 1.9 | 2 | | 0.7 |
| Indeterminate | 20 | | 3.9 | 24 | 5.8 | 3 | 0.7 | 6 | 1.6 | 13 | | 4.6 |
| Negative | 490 | | 95.9 | 380 | 91.4 | 405 | 98.8 | 352 | 96.4 | 268 | | 94.7 |
| *Missing* | | *(0)* | *(0)* | *(12)* | *(2.8)* | *(23)* | *(5.3)* | *(40)* | *(9.9)* | *(20)* | | *(6.6)* |
| Asymptomatic infection defined as positivity for at least one *Leishmania* marker. | | | | | | | | | | | | |
| ^a^ From the missed visits, 26 were planned after closure of the study  ^b^ From the missed visits 101 were planned after closure of the study  ^c^ Percentage of row column.  DAT: direct agglutination test; PCR: polymerase chain reaction; RDT: rapid diagnostic test | | | | | | | | | | | |  |
